# Quantity and quality of napping to mitigate fatigue and sleepiness among nurses working long night shifts: a prospective observational study

**DOI:** 10.1186/s40101-024-00378-z

**Published:** 2025-01-06

**Authors:** Kazuhiro Watanabe, Inaho Shishido, Yoichi M. Ito, Rika Yano

**Affiliations:** 1 Graduate School of Health Sciences, Hokkaido University, Kita 12, Nishi 5, Kita-Ku, Sapporo, Hokkaido 060-0812 Japan; 2 Faculty of Health Sciences, Hokkaido University, Kita 12, Nishi 5, Kita-Ku, Sapporo, Hokkaido 060-0812 Japan; 3 Data Science Center, Promotion Unit, Institute of Health Science Innovation for Medical Care, Hokkaido University Hospital, Kita 14, Nishi 5, Kita-Ku, Sapporo, Hokkaido 060-8648 Japan

**Keywords:** Fatigue, Long working hours, Nap, Nurses, Shift work schedule, Sleep, Sleepiness, Sleep hygiene

## Abstract

**Background:**

Napping during night shifts is a countermeasure against fatigue and sleepiness, which both impact patient safety. However, there is insufficient evidence on how nurses nap, especially concerning their napping quality. This study explored night-shift napping and its associated factors among nurses, considering napping quantity and quality, to mitigate fatigue and sleepiness.

**Methods:**

This month-long prospective observational study included 32 nurses working 16-h night shifts in a general ward. All nurses responded to questions on individual factors, while fatigue and sleepiness were checked four times during night shifts. Night-shift napping was measured using a wearable device and classified into six groups: time in bed [TIB] > 180 min and sleep efficiency [SE] ≥ 70%, TIB > 180 min and SE < 70%, TIB 120–180 min and SE ≥ 70%, TIB 120–180 min and SE < 70%, TIB < 120 min and SE ≥ 70%, and TIB < 120 min and SE < 70%.

**Results:**

Most nurses (81.2%) worked four night shifts per month, and 105 night shifts in which nurses intended to nap were analyzed. The two nap conditions (TIB 120–180 min and SE ≥ 70%, TIB > 180 min and SE ≥ 70%) were not worse than other nap conditions in fatigue and sleepiness at the end of the night shift and change in fatigue from the start to the end of the night shift. Sleep reactivity, pre-nap time on electronic devices, and prophylactic naps taken before the night shift were each the common factors related to napping for TIB ≥ 120 min and SE ≥ 70%.

**Conclusions:**

Nurses working long night shifts should consider both sufficient napping quantity and good napping quality. We suggest aiming for a TIB of at least 120 min and a SE of at least 70% to mitigate fatigue and sleepiness at the end of a night shift. Assessing sleep reactivity, pre-nap time on electronic devices, and prophylactic naps may be useful in achieving both quantity and quality effectively. Nurses and their managers should have a better understanding of napping and consider strategically taking naps during night shifts.

**Supplementary Information:**

The online version contains supplementary material available at 10.1186/s40101-024-00378-z.

## Background

Nurses work shifts to provide round-the-clock patient care [1, 2]. In recent years, the traditional three-shift system has been replaced with a two-shift system, which entails longer working hours for nurses [3, 4]. The recent global nursing shortage has also forced nurses to work longer hours and additional shifts [5, 6].

Since these shift patterns can cause insufficient or disrupted sleep [7], fatigue and sleepiness—the two most common complaints of night-shift nurses [8]—increase [9, 10]. Despite sometimes being referred to interchangeably, fatigue and sleepiness are distinct phenomena with different diagnoses and treatments [11]: while the former “refers to an overwhelming sense of tiredness, lack of energy, and a feeling of exhaustion associated with impaired physical and/or cognitive functioning;” the latter “refers to a tendency to fall asleep” [12]. Fatigue and sleepiness are associated with poor nursing performance [13, 14], an increased risk of errors during patient care [13, 14], and driving accidents after night shifts [13, 15]. Nurses and their managers should manage fatigue and sleepiness during the night shift to foster nurses’ health and patient safety.

One countermeasure to fatigue and sleepiness for night-shift workers is napping during the night shift. This countermeasure has been supported by nurses [16] and numerous positions and policy statements from federal and trade organizations [13, 17–20]. Many experimental studies suggest that napping during night shifts has more benefits than not napping [21]. Regarding night shifts among nurses, previous studies reported that the total nap duration (TND) during night shift was associated with fatigue [22, 23]. However, a systematic review focusing on napping during night shifts among nurses has not concluded how nurses can achieve night-shift napping to mitigate fatigue and sleepiness [24]. Another systematic review also reported that identifying optimal napping parameters, such as actual duration during night shifts, is challenging [25].

Meanwhile, it was reported that sleep efficiency (SE) is a more important parameter than TND for predicting sleepiness [26]. SE is one of the major objective sleep quality parameters [27, 28]. Integrating previous studies, sleep parameters of both quantity and quality may be associated with fatigue or sleepiness. Hence, it is necessary to focus on both the quantity and quality to mitigate fatigue and sleepiness in napping during night shifts.

Further, to develop countermeasures for these napping parameters of quantity and quality, the factors associated with napping should be identified. Nurses’ nap duration varies between night shifts [22]. Sleep-related problems among night-shift nurses have been divided into fixed and variable individual factors [8]. Variations in nap duration across nurses are owing to variable individual factors, such as napping environment, ways of spending breaks, and working environment [22]. There is scarce evidence not only about these environmental factors but also about what types of nurses may have these napping parameters during night shifts.

Therefore, the primary aim of this study was to examine napping among nurses working long night shifts, considering both quantity and quality, to mitigate fatigue and sleepiness. The secondary aim was to explore the factors associated with the primary aim based on nurses’ characteristics. The results are expected to provide concrete suggestions regarding napping quantity and quality during night shifts, while also contributing to nurses’ health, reducing the risk of traffic accidents when nurses drive home, and improving patient safety by mitigating nurses’ fatigue and sleepiness.

## Methods

### Design and setting

Our investigation used a prospective observational design and was conducted in accordance with the Strengthening the Reporting of Observational Studies in Epidemiology guidelines (see Additional file 1). To examine the effect of napping during the night shift, our target population was nurses who were provided with sufficient rest breaks, such as at least 2 h, as described in the guidelines of the Japanese Nursing Association [29]. However, because nurses frequently experience missed, interrupted, or delayed rest breaks due to job demands and resources [30], a survey of Japanese hospitals showed an average nap break of 1.3 h and an average nap duration of 0.9 h in two-shift work schedules in Japan [31]. Further, different environmental factors in each ward affect nurses’ total nap duration during night shifts, and TND affects fatigue [22]. Hence, to adjust for these biases and achieve our aims, our investigation was conducted over multiple night shifts in one hospital ward, which was one of the few workplaces where sufficient nap break durations were available. Further, to exclude the impact of the COVID-19 pandemic on hospitals, our research was conducted in November 2021, when the Japanese government lifted the state of emergency.

### Participants

Participants were nurses employed in a general hospital with approximately 300 beds in northern Japan. The exclusion criteria were a present or past history of sleep-related problems and current pregnancy. Sample size calculations were not performed because all nurses who fulfilled the criteria were included in the sample.

### Work conditions

The study setting was a five-department mixed ward with approximately 50 beds, where nurses engage in a two-shift system with night shifts of over 16-h—the most common shift pattern in Japan [31]. Night shifts lasted from 16:30 to 9:15 and always included four nurses, one of whom had a leadership role in addition to caring for the same number of patients as the others.

Nurses were allowed a 30-min supper break and at least a 2-h nap break, the duration of which varied depending on the situation. The nurses tried to take the longest possible nap breaks without compromising patient safety, using one of two rooms in the ward where they could control the lighting and air conditioning themselves. The first room, located in front of the nurses’ station, was a break room with a convertible sofa that could be used as a bed. The second one, located near the nurses’ station, was an informed consent room with a folding bed.

### Variables/measurement

#### Napping/activity

An activity-based sleep monitor—the MTN-221 (ACOS Co., Ltd., Iida, Japan), comparable to the Actiwatch [32]—was used to collect data without interfering with the nurses’ napping and activity. This wearable device (diameter, 27 mm; thickness, 9.1 mm; weight, 9 g) with a built-in three-axis accelerometer recorded the amount of activity and posture in six directions every two min. The agreement rates of sleep/wake states between this device and polysomnography are about 85% [33].

Nurses were required to clip it to their uniforms on the front side of the trunk only during the night shift. Data were analyzed using the Sleep Sign Act version 2.0 software (KISSEI COMTEC Co., Ltd., Matsumoto, Japan). In-bed and out-of-bed times were manually set with reference to the in-bed and out-of-bed times reported by the nurses and the data, to detect sleep/wake state and napping parameters calculated according to a previously reported algorithm of default settings [33]:

1. TIB (min): Time in bed. Duration spent in a lying posture.
2. SL (min): Sleep latency. Duration of the interval between changing postures from standing to lying and the first sleep-onset time.
3. WASO (min): Wake after sleep onset. Total duration one stays awake during the sleep onset to sleep offset interval.
4. BOL (min): Bed out latency. Duration of the interval between the last awakening time and the time of changing posture from lying to standing.
5. TND (min): Total nap duration. TIB minus SL, WASO, and BOL.
6. SE (%): Sleep efficiency. TND to TIB ratio.

The total and hourly steps between 17:00 and 9:00 the following day were calculated using the same software as an index reflecting work demands [34].

#### Fatigue

Fatigue during night shifts was measured using the *Jikaku-sho Shirabe* questionnaire developed by the Research Group of Industrial Fatigue, which is part of the Japan Society for Occupational Health. This validated questionnaire, commonly used with Japanese workers [23, 35], comprises 25 subjective fatigue symptoms rated on a five-point scale (1 = *disagree completely* to 5 = *agree strongly*). Overall scores range from 25 to 125 points, with higher scores indicating greater fatigue. Nurses completed the questionnaire four times per night shift: at the beginning, before a nap break, after a nap break, and at the end.

#### Sleepiness

Sleepiness during night shifts was measured using the Japanese version of the Karolinska Sleepiness Scale (KSS) [36, 37]. This one-dimensional instrument uses a nine-point Likert scale (1 = *very alert* to 9 = *very sleepy, fighting sleep*) and can be used repeatedly to measure the correlation with electroencephalography vigilance measurements [37].

#### Nurses’ characteristics

To explore the factors associated with night-shift napping parameters of quantity and quality, we investigated items based on previous studies, such as Spielman’s 3P model [38, 39], the Sleep Hygiene Practices Scale [40, 41], and environmental factors [22]. We defined fixed and variable individual factors [8] as nurse-related and night shift-related factors, respectively. Regarding nurse-related factors, participants completed self-administered questionnaires, which requested information such as basic attributes, before and after the investigation. Night shift-related factors, such as napping environment, ways of spending breaks, working environment, mood states, arousal level, intention to nap, and sleep-related status (which excludes sleep from naps during the night shift) were obtained during each night shift (see Additional file 2 for more detail).

### Statistical analysis

Data were presented using means (standard deviations) or frequencies (percentages). We excluded from later analyses the night shifts in which participants did not intend to nap. Pearson’s correlation analysis was performed to examine the association between continuous variables.

We examined the associations between the napping parameters to consider napping quantity and quality together. Regarding napping quantity, the TND used in previous studies of napping during night shifts [22, 23] is the typical parameter used to measure napping quantity. For napping quality, according to the National Sleep Foundation, SE is one of the appropriate indicators of objective sleep quality [28]. However, as these two indicators are very strongly correlated (*r* = 0.86, *p* < 0.001), we thought it improper to use them together. On the other hand, TIB, often used to measure sleep quantity in the past, was related to fatigue and sleepiness as well [42, 43]. Additionally, TND is the product of TIB and SE, and the correlation between TIB and SE was moderate (*r* = 0.44, *p* < 0.001). Hence, we used TIB for the napping quantity and SE for the napping quality as the target value instead of TND. Focusing on lying-down duration first, rather than sleep duration, may make it easier for nurses to practice napping.

Since most nurses (81.2%) worked four night shifts per month, we used a restricted maximum likelihood-based mixed-effects model for repeated measures (MMRM) to examine the associations between napping quantity and quality and fatigue or sleepiness. An unstructured covariance matrix was used to model the correlations among the repeated measures on different night shifts. Four time point outcome variables were set for both fatigue and sleepiness: (1) after nap breaks; (2) end of the night shift; (3) Δbefore to after nap breaks (after nap breaks minus before nap breaks); and (4) Δstart to end of the night shift (end of the night shift minus start of the night shift). As the nap timing during the night shift affects both fatigue and sleepiness [44–46], the midpoint between the first sleep-onset time and last awakening time during the TIB was used as the nap timing, with reference to the Munich Chronotype Questionnaire [47]. This nap timing was included as a covariate in all models. All continuous variables were centered at the grand mean to avoid multicollinearity.

For each outcome variable, we first conducted Model 1 using the continuous variables TIB and SE, and their interactions as fixed effects to examine the effects of both napping quantity and quality during night shifts. Second, to help interpret the quantity and quality of the napping and to confirm the details of the associations, we conducted an exploratory analysis of Model 2 that transformed TIB and SE into category variables. Grouping sleep parameters into categories is a strategy often used to associate sleep-related parameters and outcomes [26, 48–50]. Regarding TIB, we selected the cutoff points 120 and 180 min. This is because the investigated hospital’s regulation for nap breaks was at least two hours. The other reason is the guideline of the Japanese Nursing Association also recommends nap breaks of at least two hours to achieve one non-rapid eye movement (NREM)/rapid eye movement (REM) cycle based on sleep physiology [29]. Previous studies also divided the napping quantity by 120 or 180 min [23, 51–54]. According to these reasons, TIB was divided into three groups: (< 120 min, 120–180 min, and > 180 min). Regarding SE, ≥ 85% is generally considered indicative of good sleep quality [28]. However, the shorter the TIB, the more difficult it is to achieve a high SE. A calculated factor that reduces SE is the awakening duration, which includes SL, WASO, and BOL. In particular, SL does not shorten with a shorter TIB, and the relative rate of one min in the numerator increases when the TIB of the denominator becomes shorter. Hence, we considered that SE ≥ 85% could not be applied to napping during the night shift. Previous studies have also shown that the mean range of SE in napping during 16-h night shifts—the same shift pattern as in this study—is approximately 60–77% [22, 55] and no more than 85%. Hence, based on the assumption that taking a nap with a duration of one NREM/REM sleep cycle is effective [22, 29], the TND to be achieved was 85 min, which corresponds to a median of 70–100 min for the first sleep cycle duration [56]. As the SE to achieve this TND of 85 min in a TIB of 120 min was approximately 70%, we classified SE into two groups: ≥ 70% and < 70%. We combined these three groups of TIB and two groups of SE to classify the night shift into six groups, which were used as fixed effects in Model 2. The least squares means and 95% confidence intervals (CIs) for each time point of fatigue and sleepiness in each group were estimated by the Model 2. If a significant main effect of the group was found, post-hoc *t*-tests were conducted to estimate the mean difference and their 95% CIs between groups. Third, to perform a sensitivity analysis of the effects of sleep duration, we used Model 3 with the continuous variable of TND as a fixed effect.

Work demands may affect night-shift napping, fatigue, and sleepiness [30, 34, 57]. The correlation between the number of total steps, which reflects one of the work demands [34], and night shift napping was examined. The effects of work demands on fatigue and sleepiness were also examined by using the number of total steps as fixed effects alone in the above-mentioned models.

Finally, univariate analyses were conducted to explore nurse characteristics associated with napping that achieved our primary aim. We counted the number of naps that met each napping parameter identified in the above analysis over one month. Variables regarding night shifts were summarized as one-month averages for each nurse and used as night shift-related factors. The outcome variables were the rates of achieving each napping parameter per month. Nurse-related and night shift-related factors were the explanatory variables. A generalized linear model from a binomial family and a logit link function were used to estimate the odds ratios and their 95% CIs for each explanatory variable. In these explanatory analyses, pairwise deletion was used for missing data.

Significance was set at α = 0.05 since this study was exploratory. All analyses were conducted using JMP® Pro software version 17.2 (SAS Institute Inc., Cary, NC, USA) and R version 4.3.1 (R Core Team, 2023).

## Results

### Individual factors

All 32 nurses working night shifts in the ward consented to participate, without any declaring that they fulfilled the exclusion criteria. Nurse-related factors are presented in Table 1. Night shift-related factors are shown in Additional file 3.

**Table 1:** Nurse-related factors (*n* = 32)

Variables	Values
**Basic attributes**	
Age [years]: mean (*SD*)	34.6 (10.2)
Nursing experience^a^ [years]: mean (*SD*)	11.8 (8.8)
Nursing experience in the current ward^a^ [years]: mean (*SD*)	3.3 (1.6)
Sex: *n* (%)	
Female	29 (90.6)
Male	3 (9.4)
Educational level^a^: *n* (%)	
Vocational school	15 (48.4)
Junior college	3 (9.7)
University	13 (41.9)
BMI^†^: mean (*SD*)	21.1 (3.1)
Marital status^b^ (living together) [Yes]: *n* (%)	9 (30.0)
Children living together^a^ [Yes]: *n* (%)	7 (22.6)
Preschool child-rearing^a^ [Yes]: *n* (%)	3 (9.7)
Fatigue (OFER)	
Chronic Fatigue: mean (*SD*)	46.8 (15.2)
Acute Fatigue: mean (*SD*)	57.5 (19.8)
Intershift Recovery: mean (*SD*)	49.0 (21.8)
Resilience: mean (*SD*)	73.9 (9.8)
Burn Out	
Emotional exhaustion: mean (*SD*)	3.4 (0.9)
Depersonalization: mean (*SD*)	2.3 (0.8)
Decline in personal accomplishment: mean (*SD*)	3.8 (0.7)
**Sleep-related characteristics**	
Subjective mean daily sleep duration [hour]: mean (*SD*)	6.2 (1.1)
Subjective chronotype: *n* (%)	
extreme late type	3 (9.3)
moderate late type	11 (34.4)
slight late type	7 (21.9)
normal type	5 (15.6)
slight early type	0 (0.0)
moderate early type	4 (12.5)
extreme early type	2 (6.3)
Sleep reactivity (FIRST): mean (*SD*)	20.9 (6.4)
Sleep quality (PSQI): mean (*SD*)	5.4 (2.7)
poor sleep quality (total score > 5.5): *n* (%)	14 (43.8)
**Sleep-related habits**	
Alcohol intake [times/week]: mean (*SD*)	2.0 (2.3)
Caffeine intake [times/week]: mean (*SD*)	3.8 (3.0)
Exercise [times/week]: mean (*SD*)	1.3 (1.8)
Daily time spent on electronic devices [min/day]: mean (*SD*)	122.8 (82.6)
Time spent on electronic devices before bedtime [min/day]: mean (*SD*)	23.6 (18.8)
**Working environment during the investigated month**	
Number of night shifts [times]: mean (*SD*)	4.0 (0.6)
Number of days off [day]: mean (*SD*)	10.5 (1.4)
Overtime [hour]: mean (*SD*)	7.2 (3.1)

*Abbreviation*: *FIRST* Ford Insomnia Response to Stress Test, *OFER* Occupational Fatigue Exhaustion Recovery Scale, *PSQI* Pittsburgh Sleep Quality Index, *SD* standard deviation

^a^ *n* = 31, ^b^ *n* = 30

### Status of nurses’ night shifts and napping during night shifts over one month

**Fig. 1:**
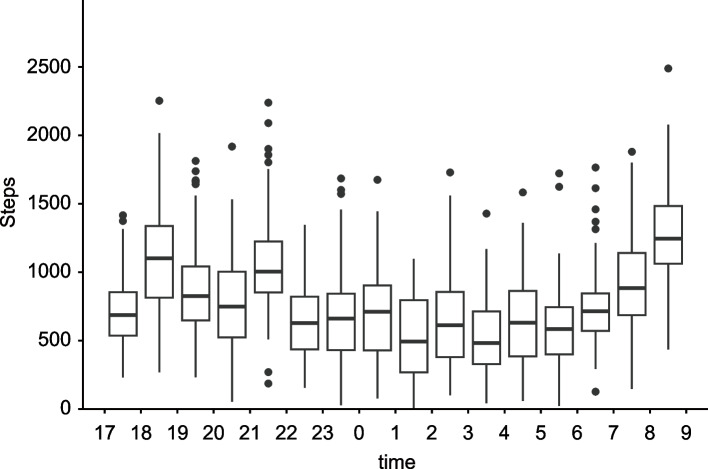
Box plots indicating the change and distribution of the number of steps taken during the night shifts The samples in this figure are 119 night shifts for one month in the investigated ward. One night shift with incomplete measurements was excluded. The steps taken during nap breaks are excluded from the calculation.

All 120 night shifts in the ward over one month were investigated, with no missing data on napping, fatigue, or sleepiness. Twenty-six (81.2%) nurses worked four night shifts per month, four (12.5%) worked three night shifts, and two (6.3%) worked two night shifts. All night shifts included nap breaks, but no napping was detected during the 12 night shifts of the three nurses who did not always nap. Two nurses differed in their intention to take naps depending on the night shift, without intention of taking naps for three night shifts. We excluded from the analyses the 15 night shifts noted above. Table 2 lists the napping parameters of the 105 night shifts wherein the 29 nurses intended to nap. Although there are five night shifts in which five nurses had short nap breaks (between 1–2 h, depending on workload), these night shifts are included in the 105 night shift used in the analysis. The steps showed a bimodal pattern, with a greater number in the evening and morning, and a particular increase from 6:00 to the end of the night shifts (Fig. 1).

**Table 2:** Night-shift napping parameters (*n* = 105)

**Variables**	Mean	*SD*	Minimum	Maximum
Time in bed (TIB) [min]	159.8	35.5	44.0	238.0
Start time of lying [h:m]	1:09	1:54	21:54	4:30
Sleep latency (SL) [min]	24.6	18.0	4.0	82.0
Start time of napping [h:m]	1:34	1:53	21:58	5:12
Total nap duration (TND) [min]	111.0	40.0	14.0	210.0
Sleep efficiency (SE) [%]	67.9	16.4	22.6	93.9
Wake after sleep onset (WASO) [min]	17.7	17.2	0.0	76.0
Frequency of awakenings during napping [times]	1.5	1.3	0.0	5.0
End time of napping [h:m]	3:43	1:44	0:42	5:48
Bed out latency (BOL) [min]	6.5	3.7	2.0	26.0
End time of lying [h:m]	3:49	1:44	0:46:	5:56

The sample units in this table are the number of night shifts. Night shifts in which nurses did not intend to nap were excluded

*Abbreviation*: *SD* standard deviation

### TIB and SE’s relationship with fatigue and sleepiness

Table 3 shows the MMRM results for Model 1 with fixed effects as TIB, SE, and their interactions of continuous variables (Table 3, Model 1). Although there were no significant main effects of TIB and interactions of TIB and SE, SE had a significant main effect on both fatigue and sleepiness at the end of the night shift. The partial regression coefficient (standard error) of SE on fatigue at the end of the night shift was −0.12 (0.06) (*p* = 0.035), while that on sleepiness at the end of the night shift was −0.03 (0.01) (*p* = 0.038).

**Table 3:** Mixed-effects model for repeated measures for fatigue and sleepiness

**Outcome symptoms** Model and fixed effects	**Time points**							
	(1) After nap breaks		(2) End of the night shift		(3) Δbefore to after nap breaks		(4) Δstart to end of the night shifts	
	*F* (*df*)	*p*	*F* (*df*)	*p*	*F* (*df*)	*p*	*F* (*df*)	*p*
**Fatigue**								
Model 1								
TIB (Continuous variable)	0.1 (1, 66.9)	.729	2.9 (1, 63.9)	.093	0.0 (1, 85.3)	.879	0.1 (1, 80.1)	.701
SE (Continuous variable)	0.0 (1, 48.7)	.837	4.7 (1, 53.3)	.035	0.3 (1, 66.9)	.580	1.4 (1, 56.8)	.243
TIB * SE	0.0 (1, 61.1)	.881	2.3 (1, 66.2)	.131	0.5 (1, 79.1)	.464	1.4 (1, 74.8)	.248
Nap Timing	0.0 (1, 60.8)	.883	0.4 (1, 59.1)	.543	8.8 (1, 69.5)	.004	0.7 (1, 69.0)	.413
Model 2								
TIB & SE group	1.3 (5, 50.7)	.260	6.0 (5, 56.0)	< .001	0.1 (5, 72.0)	.989	2.6 (5, 47.4)	.035
Nap Timing	0.1 (1, 60.6)	.750	0.0 (1, 61.8)	.824	8.2 (1, 72.2)	.005	0.4 (1, 62.7)	.529
Model 3								
TND (Continuous variable)	0.2 (1, 72.3)	.663	16.6 (1, 61.9)	< .001	0.5 (1, 71.4)	.493	4.1 (1, 69.6)	.046
Nap Timing	0.1 (1, 61.6)	.821	0.2 (1, 59.7)	.634	9.3 (1, 74.9)	.003	0.4 (1, 65.3)	.547
Model 4								
Total steps	0.0 (1, 67.0)	.864	0.0 (1, 66.3)	.971	0.0 (1, 69.5)	.858	1.0 (1, 72.7)	.323
**Sleepiness**								
Model 1								
TIB (Continuous variable)	2.4 (1, 83.8)	.123	1.6 (1, 62.8)	.209	0.6 (1, 87.7)	.429	0.5 (1, 82.9)	.477
SE (Continuous variable)	0.2 (1, 88.6)	.621	4.5 (1, 75.3)	.038	1.2 (1, 75.9)	.285	0.0 (1, 71.4)	.883
TIB * SE	0.1 (1, 76.8)	.750	0.6 (1, 70.4)	.424	0.0 (1, 78.6)	.891	0.7 (1, 75.6)	.393
Nap Timing	0.1 (1, 94.2)	.776	0.3 (1, 72.7)	.603	6.8 (1, 75.2)	.011	0.6 (1, 88.2)	.447
Model 2								
TIB & SE group	2.0 (5, 73.0)	.096	2.9 (5, 45.8)	.025	1.9 (5, 76.9)	.102	0.9 (5, 59.3)	.492
Nap Timing	0.1 (1, 89.8)	.793	0.0 (1, 56.0)	.845	6.8 (1, 82.9)	.011	0.7 (1, 78.8)	.393
Model 3								
TND (Continuous variable)	1.6 (1, 73.4)	.206	1.4 (1, 58.2)	.243	0.1 (1, 75.4)	.708	0.3 (1, 71.7)	.589
Nap Timing	0.5 (1, 96.1)	.480	0.0 (1, 55.6)	.910	9.0 (1, 82.7)	.004	0.5 (1, 81.5)	.471
Model 4								
Total steps	0.4 (1, 79.1)	.554	0.1 (1, 63.0)	.719	0.1 (1, 60.4)	.805	0.8 (1, 73.0)	.369

Night shifts in which nurses did not intend to nap were excluded. ΔBefore to after nap breaks were calculated as fatigue or sleepiness of after nap breaks minus before nap breaks. ΔStart to end of the night shifts were calculated as fatigue or sleepiness of end of the night shift minus start of the night shift. Fixed effects were analyzed using the mixed-effects model for repeated measures. The nap timing was used as a covariate

*Abbreviation*: *df* degree of freedom, *SE* sleep efficiency, *TIB* time in bed, *TND* total nap duration

### Relationship between the combined TIB and SE groups and fatigue and sleepiness

Napping during the night shift was classified by TIB and SE, which were combined into six groups. TIB > 180 min and SE ≥ 70% was appeared in 23 night shifts (21.9%), TIB > 180 min and SE < 70% was present in 12 night shifts (11.4%), TIB 120–180 min and SE ≥ 70% was present in 24 night shifts (22.9%), TIB 120–180 min and SE < 70% appeared in 31 night shifts (29.5%), TIB < 120 min and SE ≥ 70% was present in four night shifts (3.8%), TIB < 120 min and SE <70% occurred in 11 night shifts (10.5%). SE ≥ 70% was 26.7% (4/15 night shifts) when TIB < 120 min, whereas SE ≥ 70% was 43.6% (24/55 night shifts) when TIB was 120–180 min, and 65.7% (23/35 night shifts) when TIB was > 180 min.

The combined TIB and SE groups had a main effect on fatigue and sleepiness at the end of the night shift and the Δfatigue from the start to the end of the night shift (Table 3, Model 2). The results of all post-hoc *t*-tests for the three outcomes that the main effect of combined TIB and SE groups are shown in Table 4 and Additional files 4, 5, and 6. Regarding fatigue at the end of the night shift, the nap conditions of TIB < 120 min and TIB > 180 min when SE was below 70% were more fatigued than most of the other nap conditions. Even when SE was 70% or more, the nap condition of TIB < 120 min had larger Δfatigue from the start to the end of the night shift than nap conditions of TIB 120–180 min and TIB > 180 min. There were no significant differences in fatigue and sleepiness at the end of the night shift and Δfatigue from the start to the end of the night shift between TIB 120–180 min with SE ≥ 70% and TIB > 180 min with SE ≥ 70%. Furthermore, the nap condition of TIB 120–180 min with SE < 70% had higher sleepiness at the end of the night shift than that of TIB 120–180 min with SE ≥ 70%. Specifically, for TIB > 180 min, fatigue at the end of the night shift was 7.2 [1.8, 12.5] less for SE ≥ 70% than for SE < 70%. ΔFatigue from the start to the end of the night shift for TIB 120–180 min was 15.5 [3.9, 27.1] less than for TIB < 120 min, while TIB > 180 min was 12.4 [0.1, 24.7] less than for TIB < 120 min when SE was ≥ 70%. For TIB 120–180 min, sleepiness at the end of the night shift for SE ≥ 70% was 1.3 [0.3, 2.2] less than for SE < 70%.

**Table 4:**
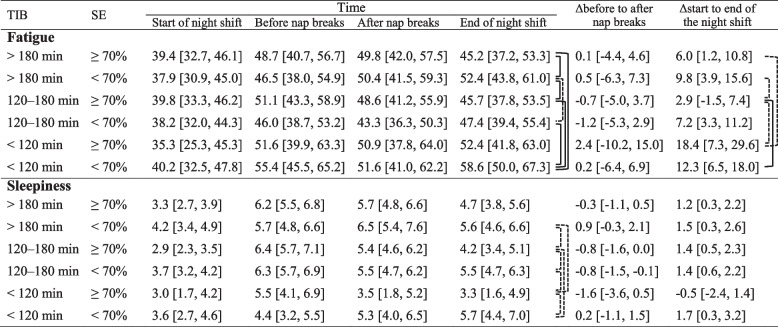
Changes and comparison in fatigue and sleepiness between combined TIB and SE groups during night shift (LS Mean [95%CI])

Night shifts in which nurses did not intend to nap were excluded. ΔBefore to after nap breaks were calculated as fatigue or sleepiness of after nap breaks minus before nap breaks. ΔStart to end of the night shifts were calculated as fatigue or sleepiness of end of the night shift minus start of the night shift. The least squares means were estimated using the mixed-effects model for repeated measures. When after nap breaks, at the end of the night shift, Δbefore to after nap breaks, and Δstart to end of the night shift, post hoc *t*-tests were conducted using their estimates between groups if there were main effects for the combined TIB and SE groups in Model 2

dot line: *p* < .05. line: *p* < .01

*Abbreviation*: CI confidence interval, LS least squares, SE sleep efficiency, TIB time in bed

Note that the means (standard deviations) of the TND for each group are as follows: TIB < 120 min and SE ≥ 70% was 87.0 (16.2) min, TIB < 120 min and SE < 70% was 39.8 (17.8) min, TIB 120–180 min and SE ≥ 70% was 129.5 (16.5) min, TIB 120–180 min and SE < 70% was 90.8 (19.4) min, TIB > 180 min and SE ≥ 70% was 160.6 (16.4) min, and TIB > 180 min and SE < 70% was 104.7 (16.2) min.

### TND’s relationship with fatigue and sleepiness

There were significant main effects of TND on fatigue at the end of the night shift and Δfatigue from the start to the end of the night shift (Table 3, Model 3). However, TND did not have any significant effect on any of the sleepiness outcomes. The partial regression coefficient (standard error) of TND on fatigue at the end of the night shift was −0.09 (0.02) (*p* < 0.001) and onΔfatigue from the start to the end of the night shift was −0.06 (0.03) (*p* = 0.046).

### Work demands’ relationship with napping, fatigue, and sleepiness

There was no correlation between total steps and TND (*r* = −0.09, *p* = 0.339) or SE (*r* = 0.06, *p* = 0.569), while the correlation between total steps and TIB was weak (*r* = −0.23, *p* = 0.018). However, the number of total steps were no main effects on fatigue or sleepiness at any time point in the Model (Table 3, Model 4).

### Factors associated with the napping that achieved the primary aim

We explored the factors associated with napping that achieved our primary aim of TIB ≥ 120 min or SE ≥ 70%. Univariate generalized linear models indicated some factors that could be associated with achieving TIB ≥ 120 min or SE ≥ 70% (Tables 5 and 6). Among them, there were three factors common to both quantity and quality. Among the nurse-related factors, sleep reactivity (Ford Insomnia Response to Stress Test score) was associated with both napping quantity and quality. Moreover, regarding the night-shift related factors, mean time spent on electronic devices before napping (e.g., individual cellphones and tablets) and the mean number of prophylactic naps taken before the night shift were associated with both napping quantity and quality.

**Table 5:** Nurse-related factors associated with TIB ≥ 120 min or SE ≥ 70% (*n* = 29)

Explanatory variables	Outcome variables			
	TIB ≥ 120 min		SE ≥ 70%	
	*OR* [95% CIs]	*p*	*OR* [95% CIs]	*p*
**Basic attributes**				
Age [years]	0.97 [0.92, 1.02]	.221	1.01 [0.98, 1.05]	.500
Nursing experience [years]	0.98 [0.92, 1.04]	.451	1.02 [0.97, 1.06]	.493
Nursing experience in the current ward^a^ [years]	1.08 [0.76, 1.51]	.672	1.28 [1.00, 1.65]	.049
Sex				
Female	0.57 [0.03, 3.35]	.583	4.90 [1.18, 33.28]	.027
Male	1.00 [Ref]		1.00 [Ref]	
Educational level^a^				
Vocational school	0.77 [0.22, 2.53]	.672	1.33 [0.57, 3.12]	.511
Junior college	1.62 [0.23, 32.74]	.663	4.31 [1.09, 21.79]	.036
University	1.00 [Ref]		1.00 [Ref]	
BMI^a^	1.07 [0.86, 1.41]	.570	1.19 [1.01, 1.44]	.039
Marital status^b^ (living together)				
Yes	0.72 [0.23, 2.36]	.572	2.50 [1.08, 6.00]	.032
No	1.00 [Ref]		1.00 [Ref]	
Children living together^a^				
Yes	1.00 [0.31, 3.90]	.995	2.23 [0.91, 5.68]	.078
No	1.00 [Ref]		1.00 [Ref]	
Preschool child-rearing^a^				
Yes	0.76 [0.17, 5.35]	.748	1.31 [0.37, 4.85]	.672
No	1.00 [Ref]		1.00 [Ref]	
Fatigue: OFER				
Chronic Fatigue	0.98 [0.94, 1.02]	.333	0.99 [0.96, 1.01]	.390
Acute Fatigue	0.97 [0.94, 1.00]	.061	0.98 [0.96, 1.00]	.078
Intershift Recovery	1.01 [0.98, 1.04]	.511	1.01 [1.00, 1.03]	.109
Resilience	1.01 [0.95, 1.06]	.824	1.02 [0.98, 1.07]	.246
Burn Out				
Emotional exhaustion	0.61 [0.31, 1.15]	.126	0.71 [0.45, 1.10]	.130
Depersonalization	0.73 [0.39, 1.41]	.344	0.62 [0.37, 1.00]	.050
Decline in personal accomplishment	0.77 [0.29, 1.74]	.561	0.94 [0.53, 1.68]	.842
**Sleep-related characteristics**				
Subjective mean daily sleep duration [hour]	1.05 [0.64, 1.81]	.854	1.16 [0.81, 1.68]	.411
Subjective chronotype				
Late type	1.12 [0.23, 4.32]	.872	1.23 [0.47, 3.20]	.671
Normal type	0.38 [0.06, 2.01]	.249	0.44 [0.10, 1.73]	.242
Early type	1.00 [Ref]		1.00 [Ref]	
Sleep reactivity: FIRST score	0.90 [0.82, 0.98]	.017	0.92 [0.86, 0.98]	.008
Sleep quality: PSQI score	0.99 [0.80, 1.22]	.916	1.00 [0.86, 1.15]	.953
poor sleep quality (total score > 5.5)	0.61 [0.19, 1.83]	.380	0.71 [0.33, 1.52]	.378
good sleep quality (total score < 5.5)	1.00 [Ref]		1.00 [Ref]	
**Sleep-related habits**				
Alcohol intake [times/week]	0.91 [0.73, 1.15]	.409	1.07 [0.91, 1.27]	.407
Caffeine intake [times/week]	1.00 [0.82, 1.20]	.967	1.12 [0.98, 1.28]	.105
Exercise [times/week]	1.41 [0.95, 2.53]	.095	0.81 [0.63, 1.01]	.061
Daily time spent on electronic devices [min/day]	1.01 [1.00, 1.02]	.133	1.00 [0.99, 1.00]	.521
Time spent on electronic devices before bedtime [min/day]	1.03 [0.99, 1.08]	.103	1.00 [0.97, 1.02]	.830
**Working environment during the investigated month**				
Number of night shifts [times]	2.29 [0.68, 8.72]	.205	1.85 [0.84, 5.48]	.136
Number of days off [day]	1.37 [0.90, 2.12]	.146	0.93 [0.69, 1.24]	.614
Overtime [hour]	0.80 [0.67, 0.95]	.009	1.03 [0.91, 1.16]	.678

The odds ratios and their 95% CIs for each explanatory variable were estimated using the generalized linear model from a binomial family and a logit link function. The outcome variables were the rates of each achieved TIB ≥ 120 min and SE ≥ 70% per month

*Abbreviation*: *CI *confidence interval, *FIRST* Ford Insomnia Response to Stress Test, *OFER* Occupational Fatigue Exhaustion Recovery Scale, *OR* odds ratio, *PSQI* Pittsburgh Sleep Quality Index, *SE* sleep efficiency, *TIB* time in bed

^a^ *n* = 28, ^b^ *n* = 27

**Table 6:** Night shift-related factors associated with TIB ≥ 120 min or SE ≥ 70% (*n* = 29)

Explanatory variables (mean value per month)	Outcome variable			
	TIB ≥ 120 min		SE ≥ 70%	
	*OR* [95% CIs]	*p*	*OR* [95% CIs]	*p*
**Napping environment**				
Illuminance [lux]	1.00 [0.99, 1.03]	.694	1.01 [1.00, 1.02]	.229
Temperature [℃]	1.70 [1.00, 3.14]	.048	1.16 [0.82, 1.65]	.404
Humidity [%]	0.83 [0.62, 1.07]	.151	0.92 [0.77, 1.10]	.381
Noise level [dB *L*_Aeq_, _napping_]	1.05 [0.92, 1.23]	.500	1.03 [0.94, 1.13]	.565
**Ways of spending breaks**				
Napping place [0: break room, 1: informed consent room]	0.30 [0.07, 1.00]	.051	0.63 [0.28, 1.41]	.265
Order of nap breaks [0: first, 1: second]	0.25 [0.03, 1.69]	.159	0.39 [0.10, 1.45]	.162
Start time of nap breaks [time (h)]	0.78 [0.47, 1.25]	.313	0.86 [0.62, 1.18]	.349
End time of nap breaks [time (h)]	1.01 [0.61, 1.68]	.959	0.86 [0.60, 1.22]	.389
Nap break duration [min]	1.06 [1.03, 1.11]	< .001	1.01 [0.99, 1.02]	.578
Time spent on electronic devices before napping [min]	0.96 [0.93, 0.99]	.004	0.98 [0.95, 1.00]	.042
Caffeine intake before nap breaks [0: No, 1: Yes]	0.94 [0.22, 4.09]	.938	1.23 [0.45, 3.39]	.683
Caffeine consumption before nap breaks [mg]	1.00 [1.00, 1.02]	.308	1.00 [1.00, 1.01]	.699
Eating before napping [0: No, 1: Yes]	0.07 [0.01, 0.40]	.003	3.16 [0.79, 14.04]	.106
Listening to music during napping [0: No, 1: Yes]	0.20 [0.04, 0.94]	.042	0.95 [0.23, 3.79]	.941
**Working environment**				
Steps before napping per hour [100steps/hour]	1.17 [0.83, 1.69]	.367	1.07 [0.85, 1.36]	.555
Number of hospitalized patients [person]	1.12 [0.90, 1.41]	.307	0.93 [0.79, 1.08]	.344
Number of patients each nurse responsible for [person]	1.86 [0.89, 4.13]	.102	0.84 [0.50, 1.39]	.492
Have a leadership role [0: No, 1: Yes]	0.26 [0.04, 1.62]	.145	3.51 [0.90, 14.76]	.071
Event occurrence [0: No, 1: Yes]	0.09 [0.01, 0.66]	.017	1.41 [0.36, 5.56]	.618
**Mood States (POMS2)**				
AH (anger–hostility)	0.92 [0.79, 1.08]	.293	0.94 [0.83, 1.06]	.312
CB (confusion-bewilderment)	0.87 [0.69, 1.10]	.244	0.91 [0.76, 1.08]	.271
DD (depression–dejection)	0.80 [0.63, 1.03]	.080	0.89 [0.72, 1.08]	.240
FI (fatigue-inertia)	0.98 [0.84, 1.15]	.766	0.94 [0.84, 1.05]	.278
TA (tension–anxiety)	0.88 [0.74, 1.03]	.108	0.94 [0.83, 1.06]	.295
VA (vigor-activity)	0.96 [0.74, 1.27]	.762	1.12 [0.93, 1.37]	.243
F (friendliness)	0.88 [0.66, 1.15]	.347	1.10 [0.92, 1.33]	.311
Total mood disturbance	0.97 [0.93, 1.02]	.207	0.98 [0.94, 1.01]	.145
**Arousal level (KSS)**				
Start of the night shift	1.45 [0.86, 2.73]	.169	0.73 [0.51, 1.03]	.072
Before nap breaks	1.63 [1.01, 2.66]	.043	1.08 [0.77, 1.53]	.662
Change between start of the night shift to before nap breaks	1.14 [0.75, 1.78]	.535	1.35 [1.00, 1.84]	.051
**Intention to take napping** [0: deep sleep, 1: light sleep]	1.00 [0.25, 4.62]	.999	0.36 [0.12, 1.01]	.053
**Sleep-related status**				
Start time of main sleep before the night shift [time (h)]	1.58 [0.98, 2.76]	.060	1.03 [0.77, 1.39]	.828
End time of main sleep before the night shift [time (h)]	1.05 [0.79, 1.39]	.744	1.04 [0.86, 1.27]	.688
Main sleep duration before the night shift [hour]	0.82 [0.56, 1.17]	.272	1.04 [0.81, 1.34]	.740
Prophylactic nap [0: No, 1: Yes]	0.19 [0.04, 0.78]	.022	2.91 [1.05, 8.45]	.040
Total sleep duration before the night shift [hour]	0.58 [0.35, 0.89]	.012	1.15 [0.89, 1.51]	.280
Last awakening time before the night shift [time (h)]	0.81 [0.58, 1.08]	.156	1.45 [1.15, 1.89]	.001
Awakening duration until nap breaks [hour]	1.04 [0.82, 1.34]	.762	0.73 [0.58, 0.89]	.001

The odds ratios and their 95% CIs for each explanatory variable were estimated using the generalized linear model from a binomial family and a logit link function. The outcome variables were the rates of each achieved TIB ≥ 120 min and SE ≥ 70% per month. Explanatory variables regarding night shifts were summarized as one-month averages for each nurse, used as night shift-related factors. Categorical variables were also transformed into binary dummy variables of 0 or 1 and summarized in the same way and used as continuous variables

*Abbreviation*: *CI* confidence interval, *dB L*_Aeq, napping_ decibel equivalent A-weighted sound pressure level during napping, *KSS* Karolinska Sleepiness Scale, *OR* odds ratio, *POMS2* Profile of Mood States Second edition, *SE* sleep efficiency, *TIB* time in bed

## Discussion

Fatigue and sleepiness, the two most common complaints among nurses working night shifts [8], are distinct phenomena that should be distinguished [11] and managed. Although one of the recommended countermeasures to mitigate fatigue and sleepiness during night shifts is napping [13, 54], a systematic review showed that the effects of napping on these problems were inconclusive [24]. The primary aim of this study was to examine napping through a novel approach, considering both napping quantity and quality to mitigate fatigue and sleepiness among nurses working long night shifts. Our results showed that the effects of differences in nap conditions on fatigue and sleepiness are complex. The exploratory analyses also showed that the nap conditions of TIB ≥ 120 min and SE ≥ 70% were not worse than other nap conditions in fatigue and sleepiness at the end of the night shift and Δfatigue from the start to the end of the night shift. These results support that both quantity and quality are important even for napping during night shifts, as stated in previous studies on sleep [58]. Therefore, nurses can mitigate fatigue and sleepiness at the end of the night shift when they first lie down for at least 120 min and further achieve high-quality SE napping at least 70%, regardless of nap timing.

### Relationship between napping, fatigue, and sleepiness during the night shift

It may be noteworthy to focus on achieving a duration of at least 120 min. Previous studies reported that nurses who napped for ≥ 120 min during night shifts had lower cumulative fatigue and recovery from fatigue than those who napped for < 120 min [23, 52, 53]. The Japanese Nursing Association’s guidelines also recommend nap breaks of at least two hours [29]. Our results align with these previous studies. Additionally, a longer TIB is needed to achieve napping with higher SE. There was a moderate correlation between TIB and SE, and the exploratory analysis also showed that achieving SE ≥ 70% was easier when TIB was longer. However, a longer TIB did not significantly reduce fatigue and sleepiness. Similar findings have been reported in studies conducted in Brazil, where long naps during night shifts are routine. Those studies showed that naps > 3 h were not as effective for recovery after work as were 2–3-h naps [52, 53].

In contrast, napping with higher SE, indicating better napping quality, mitigated fatigue and sleepiness at the end of the night shift. This result does not conflict with the fact that good sleep quality has positive effects [27]. Additionally, if nurses achieve TIB ≥ 120 min and further achieve SE ≥ 70%, they may obtain at least one NREM/REM sleep cycle including slow wave sleep—the deepest NREM sleep—which plays an important role in energy recovery [56, 59]. Thus, we conclude that sufficient quantity and high-quality napping have benefits for nurses to mitigate fatigue and sleepiness.

Napping benefits not only nurses but also patients. Regarding the assumptions, the participating nurses were engaged in long night shifts (from 16:30 to 9:15), which were associated with fatigue and sleepiness [9, 10]. Fatigue and sleepiness, which peak at the end of the night shift without countermeasures [10, 60–62], impair nurses’ cognitive function and performance [63, 64]. In addition, nurses become busier in the early morning because they must provide care for patients who wake up. This is supported by our results that the number of total steps, indicating work demands [34], increased considerably from 6:00 a.m. to the end of the night shift. Thus, nurses face a higher risk of errors, such as medication administration errors and needlestick injuries, toward the end of a night shift. Previous studies showed that fatigue and other performance deficits in night-shift workers in the early morning were linked with major disasters [21], with more incidents during night than day shifts [65]. Our results showed that SE ≥ 70% reduced the KSS score by more than one point at the end of the night shift compared to SE < 70% when TIB was 120–180 min. A systematic review of sleepiness while driving reported a 1.4–1.9 times increased risk and a 5.4 times increased odds of lane deviation per one-point increase in KSS score [66]. If this is true for nurses, it may lead to errors in patient care. Napping with TIB ≥ 120 min and SE ≥ 70% during night shifts may prevent these errors and accidents, contributing to safer nursing.

Additionally, the difference in sleepiness at the end of the night shift can also reduce the risk of nurses getting into traffic accidents while driving home. Night-shift nurses are at a greater risk of dozing off while driving, driving off the road, or being involved in a car accident compared to nurses who work other shifts [15, 67]. Reducing these risks can enhance public safety.

TND was associated with fatigue but not sleepiness. A previous study showed that only SE in main sleep predicted sleepiness among sleep parameters, but not total sleep time [26]. Thus, SE may be the ideal napping parameter, as it is strongly associated with sleepiness. We cannot conclude why only SE was associated with sleepiness rather than TND and TIB, despite the strong correlation between SE and TND. As SE is a complex index of TND, TIB, SL, WASO, and BOL, further research is necessary to determine why SE is associated with sleepiness.

The number of total steps, one of the indicators of work demands [34], was not associated with either fatigue or sleepiness in this study. This supports that fatigue and sleepiness at the end of a night shift were mitigated by napping with sufficient quantity and quality.

Therefore, we propose using TIB and SE as target values instead of TND. As a first step, we recommend that nurses first aim to have a TIB of at least 120 min.

### Possible Factors Related to Napping with TIB ≥ 120 min and SE ≥ 70%

Exploring the factors associated with TIB ≥ 120 min and SE ≥ 70% revealed three factors that were commonly associated. The first one is sleep reactivity, which characterizes the degree to which stress exposure disrupts sleep, resulting in difficulty falling and staying asleep [68]. A trend similar to our results has been reported by Kalmbach et al. [68], who found that people with high sleep reactivity have low SE during their main sleep. Further, workers with high sleep reactivity had over five times the odds of developing shift work disorder compared to those with low sleep reactivity after transitioning to rotating shifts [69]. Therefore, this factor could be an important individual attribute for nurses working night shifts.

The second factor is the time spent using electronic devices before napping. The use of electronic devices during limited nap breaks not only reduces the duration to nap but also prolongs the time needed to fall asleep and increases alertness [70, 71]. A previous study found that the time spent on electronic devices was associated with TND [22]. The timing of use must be carefully considered.

The third factor is a prophylactic nap taken before the night shift, which has been reported as effective in lowering sleepiness during night shifts [54]. Our results showed that nurses who did not tend to take a prophylactic nap found it easier to achieve TIB ≥ 120 min. This may be because not taking a prophylactic nap before a night shift increases sleep pressure based on the two-process model [72, 73]. On the contrary, it is difficult to explain why nurses who took a prophylactic nap found it easier to achieve high-quality napping (SE ≥ 70%). Since our results used the one-month averages for each nurse, further studies should examine a prophylactic nap of each night shift in more detail, including individual circadian rhythm phases [74].

### Clinical implications

We recommend that night-shift naps involve a TIB of at least 120 min and an SE of at least 70%. Assessing sleep reactivity, pre-nap time on electronic devices, and prophylactic naps taken before the night shift may be useful in achieving effective napping quantity and quality. Additionally, nurse managers should implement hospital regulations to support nurses’ napping habits, and nurses should fully utilize the full nap break.

As previously reported [25, 75], it is important to note that nurses also must be aware that long naps during night shifts do not effectively reduce fatigue or sleepiness after nap breaks. Our results also showed that neither the quantity nor the quality of night-shift naps impacted fatigue and sleepiness after nap breaks, likely because of sleep inertia—defined as a brief period of decreased cognitive function or performance immediately after waking, which can temporarily obscure the recuperative effects of sleep [21]. Therefore, nurses should ensure that they have as much time as possible to recover from sleep inertia before restarting work.

### Strengths and limitations

One strength of this study is that it investigated all night shifts in a hospital ward for one month. Further, there were no deficits in napping parameters, fatigue, or sleepiness. However, there are some limitations. First, we showed that napping during night shifts had the best effect when TIB was at least 120 min and SE was at least 70%, but the effects of napping during night shifts on fatigue and sleepiness could not be fully explained by using TND, TIB, and SE. Second, selection bias may have been present. Our results were limited to one ward, and nurses who did not nap were excluded from the analysis. Third, napping was measured using wearable devices only, and sleep depth was not considered. Fourth, the effects of napping during night shifts were limited to its effects on fatigue and sleepiness, while its effects on other outcomes, such as performance and accidents, remain unclear. Hence, it is impossible to generalize our results to all shift workers. To establish the evidence for proper napping, validation involving other wards and hospitals, shift patterns, and different outcomes is required for large samples.

## Conclusion

Our results highlight the importance of considering both sufficient napping quantity and high napping quality to mitigate fatigue and sleepiness during long night shifts. Specifically, we suggest that those aiming for TIB at least 120 min and SE at least 70% can experience mitigating fatigue and sleepiness at the end of their night shifts. This napping may be proper for nurses working 16-h night shifts, if their ineffectiveness directly after a nap is considered. It may be efficient to assess sleep reactivity, time spent on electronic devices before napping, and prophylactic naps taken before the night shift to achieve both sufficient napping quantity and high napping quality. Nurses and their managers should have a better understanding of napping and its effects during night shifts and strategically adjust napping to maximize nurses’ health and patient safety on night shifts.

## Supplementary Information

Additional file 1. STROBE statement checklist.

Additional file 2. Details of methods to explore factors related to napping during night shifts.

Additional file 3. Night shift-related factors.

Additional file 4. Comparison of fatigue at the end of the night shift between combined TIB and SE groups.

Additional file 5. Comparison of sleepiness at the end of the night shift between combined TIB and SE groups.

Additional file 6. Comparison of Δfatigue from start to end of the night shift between combined TIB and SE groups.

## Data Availability

The datasets generated during and/or analyzed during the current study are not publicly available due to nurses participating in this study did not agree for all their data to be shared publicly. However, it may be possible to provide some of the data that is not privacy-related in consultation with the authors on a reasonable request.

## Abbreviations

BOL: Bed out latency
CI: Confidence interval
KSS: Karolinska Sleepiness Scale
MMRM: Mixed-effects model for repeated measures
NREM: Non-rapid eye movement
REM: Rapid eye movement
SE: Sleep efficiency
SL: Sleep latency
TIB: Time in bed
TND: Total nap duration
WASO: Wake after sleep onset
