# Supplementary material for: Quantity and quality of napping to mitigate fatigue and sleepiness among nurses working long night shifts: a prospective observational study
Source: J Physiol Anthropol. 2025 Jan 6;44:1. doi: 10.1186/s40101-024-00378-z (PMC11702087; doi:10.1186/s40101-024-00378-z)
Supplement: Supplementary file 1 — Additional file 1. STROBE statement checklist. [file 40101_2024_378_MOESM1_ESM.docx]

**Additional file 1** STROBE statement checklist

|  | Item No. | Recommendation | Page  No. | Relevant text from manuscript |
| --- | --- | --- | --- | --- |
| **Title and abstract** | 1 | (*a*) Indicate the study’s design with a commonly used term in the title or the abstract | Manuscript, page 1–3 | Title and Abstract |
|  |  | (*b*) Provide in the abstract an informative and balanced summary of what was done and what was found | Manuscript, page 2, 3 | Abstract |
| Introduction | | | |  |
| Background/rationale | 2 | Explain the scientific background and rationale for the investigation being reported | Manuscript, pages 3–5 | Background |
| Objectives | 3 | State specific objectives, including any prespecified hypotheses | Manuscript, pages 3–5 | Background |
| Methods | | | |  |
| Study design | 4 | Present key elements of study design early in the paper | Manuscript, page 5 | Methods-Design and Setting |
| Setting | 5 | Describe the setting, locations, and relevant dates, including periods of recruitment, exposure, follow-up, and data collection | Manuscript, pages 5, 6 | Methods-Design and Setting, Work Conditions |
| Participants | 6 | (*a*) *Cohort study*—Give the eligibility criteria, and the sources and methods of selection of participants. Describe methods of follow-up  *Case-control study*—Give the eligibility criteria, and the sources and methods of case ascertainment and control selection. Give the rationale for the choice of cases and controls  *Cross-sectional study*—Give the eligibility criteria, and the sources and methods of selection of participants | Manuscript, page 5, 6 | Methods-Participants |
|  |  | (*b*) *Cohort study*—For matched studies, give matching criteria and number of exposed and unexposed  *Case-control study*—For matched studies, give matching criteria and the number of controls per case | N/A |  |
| Variables | 7 | Clearly define all outcomes, exposures, predictors, potential confounders, and effect modifiers. Give diagnostic criteria, if applicable | Manuscript, pages 6–8  Additional file 2 | Methods-Variables/Measurement  Additional file 2 |
| Data sources/ measurement | 8* | For each variable of interest, give sources of data and details of methods of assessment (measurement). Describe comparability of assessment methods if there is more than one group | Manuscript, pages 6–8  Additional file 2 | Methods-Variables/Measurement  Additional file 2 |
| Bias | 9 | Describe any efforts to address potential sources of bias | Manuscript, page 5 | Methods-Design and Setting |
| Study size | 10 | Explain how the study size was arrived at | Manuscript, page 5, 6 | Methods-Participants |

Continued on next page

| Quantitative variables | 11 | Explain how quantitative variables were handled in the analyses. If applicable, describe which groupings were chosen and why | Manuscript, pages 8–11 | Methods- Statistical Analysis |
| --- | --- | --- | --- | --- |
| Statistical methods | 12 | (*a*) Describe all statistical methods, including those used to control for confounding | Manuscript, pages 8–11 | Methods- Statistical Analysis |
|  |  | (*b*) Describe any methods used to examine subgroups and interactions | Manuscript, pages 8–11 | Methods- Statistical Analysis |
|  |  | (*c*) Explain how missing data were addressed | Manuscript, pages 8–11 | Methods- Statistical Analysis |
|  |  | (*d*) *Cohort study*—If applicable, explain how loss to follow-up was addressed  *Case-control study*—If applicable, explain how matching of cases and controls was addressed  *Cross-sectional study*—If applicable, describe analytical methods taking account of sampling strategy | Manuscript, pages 8–11 | Methods- Statistical Analysis |
|  |  | (*e*) Describe any sensitivity analyses | N/A |  |
| Results | | | | |
| Participants | 13* | (a) Report numbers of individuals at each stage of study—eg numbers potentially eligible, examined for eligibility, confirmed eligible, included in the study, completing follow-up, and analysed | Manuscript, pages 12 | Results |
|  |  | (b) Give reasons for non-participation at each stage | Manuscript, pages 12 | Results |
|  |  | (c) Consider use of a flow diagram | N/A |  |
| Descriptive data | 14* | (a) Give characteristics of study participants (eg demographic, clinical, social) and information on exposures and potential confounders | Manuscript, page 32, 33 | Table 1 |
|  |  | (b) Indicate number of participants with missing data for each variable of interest | Manuscript, page 12, 32, 33  Additional file 3 | Results  Table 1  Additional file 3 |
|  |  | (c) *Cohort study*—Summarise follow-up time (eg, average and total amount) | Manuscript, page 12 | Results |
| Outcome data | 15* | *Cohort study*—Report numbers of outcome events or summary measures over time | Manuscript, pages 12–16, 33, 36  Additional file 4, 5, 6 | Results  Table 2, 4  Additional file 4, 5, 6 |
|  |  | *Case-control study—*Report numbers in each exposure category, or summary measures of exposure | N/A |  |
|  |  | *Cross-sectional study—*Report numbers of outcome events or summary measures | Manuscript, pages 12–16, 34, 37  Additional file 4, 5, 6 | Results  Table 2, 4  Additional file 4, 5, 6 |
| Main results | 16 | (*a*) Give unadjusted estimates and, if applicable, confounder-adjusted estimates and their precision (eg, 95% confidence interval). Make clear which confounders were adjusted for and why they were included | Manuscript, pages 13–16, 38–41  Additional file 4, 5, 6 | Results  Table 5, 6  Additional file 4, 5, 6 |
|  |  | (*b*) Report category boundaries when continuous variables were categorized | Manuscript, pages 13–16 | Results |
|  |  | (*c*) If relevant, consider translating estimates of relative risk into absolute risk for a meaningful time period | N/A |  |

Continued on next page

| Other analyses | 17 | Report other analyses done—eg analyses of subgroups and interactions, and sensitivity analyses | Manuscript, pages 15 | Results |
| --- | --- | --- | --- | --- |
| Discussion | | | | |
| Key results | 18 | Summarise key results with reference to study objectives | Manuscript, page 16, 17 | Discussion |
| Limitations | 19 | Discuss limitations of the study, taking into account sources of potential bias or imprecision. Discuss both direction and magnitude of any potential bias | Manuscript, pages 21 | Discussion-Strengths and Limitations |
| Interpretation | 20 | Give a cautious overall interpretation of results considering objectives, limitations, multiplicity of analyses, results from similar studies, and other relevant evidence | Manuscript, pages 16–20 | Discussion |
| Generalisability | 21 | Discuss the generalisability (external validity) of the study results | Manuscript, pages 21 | Discussion-Strengths and Limitations |
| Other information | |  | | |
| Funding | 22 | Give the source of funding and the role of the funders for the present study and, if applicable, for the original study on which the present article is based | Manuscript, pages 23 | Declarations- Funding |

*Give information separately for cases and controls in case-control studies and, if applicable, for exposed and unexposed groups in cohort and cross-sectional studies.

**Note:** An Explanation and Elaboration article discusses each checklist item and gives methodological background and published examples of transparent reporting. The STROBE checklist is best used in conjunction with this article (freely available on the Web sites of PLoS Medicine at http://www.plosmedicine.org/, Annals of Internal Medicine at http://www.annals.org/, and Epidemiology at http://www.epidem.com/). Information on the STROBE Initiative is available at www.strobe-statement.org.
