# Supplementary material for: Quantity and quality of napping to mitigate fatigue and sleepiness among nurses working long night shifts: a prospective observational study
Source: J Physiol Anthropol. 2025 Jan 6;44:1. doi: 10.1186/s40101-024-00378-z (PMC11702087; doi:10.1186/s40101-024-00378-z)
Supplement: Supplementary file 2 — Additional file 2. Details of methods to explore factors related to napping during night shifts. [file 40101_2024_378_MOESM2_ESM.docx]

**Additional file 2** Details of methods to explore factors related to napping during night shifts

| Variables |  | Measurement items or way or questionnaire | Notes | Reliability and validity |
| --- | --- | --- | --- | --- |
| **Nurse-related Factors** | |  |  |  |
| Basic Attributes | Demographic Data | Age, years of nursing experience, years of nursing experience in the current ward, sex, educational level, present or past history of sleep-related problems, marital status (living together), children living together, preschool child-rearing, and current pregnancy. |  |  |
|  | Fatigue | The Japanese version of the Occupational Fatigue Exhaustion Recovery Scale (OFER) consists of 3 subscales: Chronic fatigue; Acute fatigue; Intershift recovery. | 15 items with seven-point Likert scale (0 = *completely disagree* to 6 = *completely agree*).  Standardized scores (range: 0–100) for each factor were calculated using the following formula:  Sum of the item scores each factor ÷ 30 × 100.  Chronic and acute fatigue, higher standardized score indicates higher fatigue.  Intershift recovery, higher standardized score indicates better recovery. | Acceptable reliability and validity were shown [76]. |
|  | Resilience | The Japanese Version of the Resilience Scale for Nurses. | 22 items with five-point Likert scale (1 = *No* to 5 = *Yes*).  The total score was calculated, ranging from 22 to 110.  The higher score indicates higher resilience. | Acceptable reliability and validity were shown [77]. |
|  | Burn Out | The Japanese Burnout Scale that was based on Maslach Burnout Inventory consists of 3 subscales: Emotional exhaustion; Depersonalization; Decline in personal accomplishment. | 17 items with five-point scale (1 = *Do not feel* to 5 = *Everyday*).  Average scores for each factor were calculated.  The higher score indicates stronger symptoms. | Acceptable reliability and validity were shown [78, 79]. |
| Sleep-related characteristics | Subjective mean daily sleep duration | Subjective mean daily sleep duration. |  |  |
|  | Subjective chronotype | “Which of the types describes yourself?” | 9 responses (extreme late, moderate late, slight late, normal, slight early, moderate early, extreme early).  Nurses were reclassified into three groups (late/normal/early). | This self-reported chronotype based on a single question has shown to be in excellent agreement with quantitatively assessed chronotype based on sleep time in the Munich Chrono Type Questionnaire [47]. |
|  | Sleep reactivity | Ford Insomnia Response to Stress Test (FIRST). | This is a self-rating score measuring the likelihood of the occurrence of sleep disturbances in response to commonly experienced stressful situations [80].  9 items with four-point Likert scale (1 = *not likely* to 4 = *very likely*).  The higher score indicates higher sleep reactivity. | Acceptable validity was shown [81]. |
|  | Sleep quality | The Japanese version of the Pittsburgh Sleep Quality Index (PSQI) comprises seven components: subjective sleep quality, sleep latency, sleep duration, sleep efficiency, sleep disturbances, sleep medication use, and daytime dysfunction. | 19 items.  The global score (range: 0–21) were calculated, and the higher score indicates the poorer sleep quality.  Nurses were divided into two categories, good sleep quality and poor sleep quality, with a global score of 5.5 as the cutoff.  This questionnaire was answered after the investigation. | Acceptable reliability and validity were shown [82, 83]. |
| Sleep-related Habits |  | Frequency of alcohol or caffeine intake, frequency of exercise, and daily times spent on electronic devices (e.g., cellular phone, smartphone, tablet) during the day and before bedtime. |  |  |
| Working environment during the investigated month |  | Number of night shifts, number of days off, and overtime hours. | These items were answered after the investigation. |  |
| **Night shift-related Factors** | |  |  |  |
| Napping environment (mean or median values corresponding to the napping) | Illuminance (median) | Ondotori (T&D Corporation, Matsumoto, Japan).  Measurements were recorded every 2 min on a wagon (65 cm above the floor). | Light intensity with a range of 0–130 Klux with a minimum resolution of 0.01 lux. | Accuracy of 5% reading. |
|  | Temperature  (mean) |  | The sensor included a thermistor measuring temperature within a range of -25–70 °C with a resolution of 0.1 °C. | Accuracy of ±0.3 °C. |
|  | Humidity  (mean) |  | The polymer resistance humidity sensor had a range of 0–99%RH, and a resolution of 0.1%RH. | Accuracy of ±2.5%RH. |
|  | Noise  (Equivalent sound level) | SL-1373SD (CUSTOM Corporation, Tokyo, Japan).  Data of fast and A-weighted sound level were recorded every second on the same wagon. | Measurement range of 30–130 dB. Frequency range of 31.5 Hz to 8 kHz.  A resolution of 0.1 dB.  Equivalent sound level (dB *L*_Aeq_) was calculated. | Accuracy ±1.4 dB. |
| Ways of spending breaks |  | Napping place, order of nap breaks, start and end time of nap breaks, in-bed and out-of-bed times, time spent on electronic devices (e.g., cellular phone, smartphone, tablet) before napping, caffeine intake before nap breaks and their details, eating before napping, and listening to music during napping. | To quantify the Caffeine consumption before nap breaks, product-specific values were utilized for items with available information. Conversely, for those lacking explicit details, calculations were derived from the Standard Tables of Food Composition in Japan 2020 [84]. |  |
| Working environment |  | Number of hospitalized patients, number of patients each nurse responsible for, play the role of night shift leader, event occurrence. |  |  |
| Mood states |  | The Profile of Mood States Second edition (POMS2) adult short Japanese version consists of seven mood components: anger–hostility (AH), confusion–bewilderment (CB), depression–dejection (DD), fatigue–inertia (FI), tension–anxiety (TA), vigor–activity (VA), and friendliness (F). | Based on previous studies [85, 86], the response instructions were changed from the conventional “last week” to “right now.”  35 items with five-point scale (0 = *not at all* to 4 = *extremely*).  Total scores for the seven components were calculated.  Additionally, the total mood disturbance (TMD) was also calculated:  TMD = AH + CB + DD + FI + TA – VA.  The higher scores indicate stronger symptoms.  These items were answered before the nap breaks. | Acceptable reliability and validity were shown [87]. |
| Arousal level |  | The Japanese version of the Karolinska Sleepiness Scale. | A single item with nine-point Likert scale (1 = *extremely alert* to 9 = *very sleepy, great effort to keep awake, fighting sleep*).  Change arousal level between start of the night shift to before the nap breaks were calculated. | Acceptable reliability and validity were shown [36, 37]. |
| Intention to nap |  | “What intention did you have regarding napping during the night shift—deeply, lightly, or not at all?” | This item was answered after the nap breaks. |  |
| Sleep-related status (which excludes sleep from naps during the night shift) |  | Start and end time of main sleep before the night shift, take a prophylactic nap before the night shift (start and end times of their napping). | Awakening duration until in-bed times of napping during the night shift was calculated as an indicator of homeostatic sleep pressure [88, 89].  (These items were answered at the start of the night shift. |  |

**Abbreviation.** RH = relative humidity, dB = decibel.

**References**

36. Åkerstedt T, Gillberg M. Subjective and objective sleepiness in the active individual. Int J Neurosci. 1990;52:29–37. doi: 10.3109/00207459008994241.

37. Kaida K, Takahashi M, Åkerstedt T, Nakata A, Otsuka Y, Haratani T, et al. Validation of the Karolinska sleepiness scale against performance and EEG variables. Clin Neurophysiol. 2006;117:1574–81. doi: 10.1016/j.clinph.2006.03.011.

47. Roenneberg T, Wirz-Justice A, Merrow M. Life between clocks: daily temporal patterns of human chronotypes. J Biol Rhythms. 2003;18:80–90. doi: 10.1177/0748730402239679.

76. Yamaguchi S, Sato M, Sumi N, Ito YM, Winwood PC, Yano R. Psychometric properties of the Japanese version of the Occupational Fatigue Exhaustion Recovery Scale among shift-work nurses. J Occup Health. 2022;64:e12325. doi: 10.1002/1348-9585.12325.

77. Ihara H, Ogata H, Inuzuka A, Ohta N, Nagai T, Mizuno M. Development and psychometric validation of the resilience scale for nurses. Jpn J Gen Hosp Psychiatry. 2010;22: 210–20. doi: 10.11258/jjghp.22.210.

78. Kubo M. The factorial and construct validity of the Japanese burnout scale. J Sci Labour. 2007;83:39–53. (in Japanese)

79. Kubo M. The factorial and construct validity of the Japanese Burnout Scale among service workers. Jpn J Psychol. 2014;85:364–72. (in Japanese) doi: 10.4992/jjpsy.85.13214.

80. Drake C, Richardson G, Roehrs T, Scofield H, Roth T. Vulnerability to stress-related sleep disturbance and hyperarousal. Sleep. 2004;27:285–91. doi: 10.1093/sleep/27.2.285.

81. Nakajima S, Okajima I, Sasai T, Kobayashi M, Furudate N, Drake CL, et al. Validation of the Japanese version of the Ford Insomnia Response to Stress Test and the association of sleep reactivity with trait anxiety and insomnia. Sleep Med. 2014;15:196–202. doi: 10.1016/j.sleep.2013.09.022.

82. Buysse DJ, ReynoldsIII CF, Monk TH, Berman SR, Kupfer DJ. The Pittsburgh Sleep Quality Index: a new instrument for psychiatric practice and research. Psychiatry Res. 1989;28:193–213. doi: 10.1016/0165-1781(89)90047-4.

83. Doi Y, Minowa M, Uchiyama M, Okawa M, Kim K, Shibui K, et al. Psychometric assessment of subjective sleep quality using the Japanese version of the Pittsburgh Sleep Quality Index (PSQI-J) in psychiatric disordered and control subjects. Psychiatry Res. 2000;97:165–72. doi: 10.1016/s0165-1781(00)00232-8.

84. Ministry of Education, Culture, Sports, Science, and Technology of Japan: The standard tables of food composition in Japan 2020. <https://www.mext.go.jp/content/20201225-mxt_kagsei-mext_01110_012.xlsx> (2020). Accessed 10 Sep. 2024.

85. Corchs F, Nutt DJ, Hood S, Bernik M. Serotonin and sensitivity to trauma-related exposure in selective serotonin reuptake inhibitors-recovered posttraumatic stress disorder. Biol Psychiatry. 2009;66:17–24. doi: 10.1016/j.biopsych.2009.01.031.

86. Kenttä G, Hassmén P, Raglin JS. Mood state monitoring of training and recovery in elite kayakers. Eur J Sport Sci. 2006;6:245–53. doi: 10.1080/17461390601012652.

87. Heuchert JP, McNair DM. Profile of Mood States 2nd Edition™ (POMS). APA PsycTests. doi: 10.1037/t05057-000.

88. Zion N, Shochat T. Let them sleep: the effects of a scheduled nap during the night shift on sleepiness and cognition in hospital nurses. J Adv Nurs. 2019;75:2603–15. doi: 10.1111/jan.14031.

89. Ficca G, Axelsson J, Mollicone DJ, Muto V, Vitiello MV. Naps, cognition and performance. Sleep Med Rev. 2010;14:249–58. doi: 10.1016/j.smrv.2009.09.005.
