# Supplementary material for: Quantity and quality of napping to mitigate fatigue and sleepiness among nurses working long night shifts: a prospective observational study
Source: J Physiol Anthropol. 2025 Jan 6;44:1. doi: 10.1186/s40101-024-00378-z (PMC11702087; doi:10.1186/s40101-024-00378-z)
Supplement: Supplementary file 3 — Additional file 3. Night shift-related factors. [file 40101_2024_378_MOESM3_ESM.docx]

**Additional file 3** Night shift-related factors (*n* = 105)

| **Variables** | **Values** |
| --- | --- |
| **Napping environment** |  |
| Illuminance [lux]: mean (*SD*) | 9.6 (48.9) |
| Temperature [℃]: mean (*SD*) | 24.7 (1.2) |
| Humidity [%]: mean (*SD*) | 41.4 (2.6) |
| Noise level [dB *L*_Aeq_, _napping_]: mean (*SD*) | 38.6 (5.2) |
| **Ways of spending breaks** |  |
| Napping place: *n* (%) |  |
| Break room | 51 (48.6) |
| Informed consent room | 54 (51.4) |
| Order of nap breaks: *n* (%) |  |
| First | 54 (51.4) |
| Second | 51 (48.6) |
| Start time of nap breaks [h:m]: mean (*SD*) | 0:58 (1:53) |
| End time of nap breaks [h:m]: mean (*SD*) | 3:57 (1:44) |
| Nap break duration [min]: mean (*SD*) | 178.7 (39.6) |
| Time spent on electronic devices before napping [min]: mean (*SD*) | 19.2 (20.2) |
| Caffeine intake before nap breaks [Yes]: *n* (%) | 50 (47.6) |
| Caffeine consumption before nap breaks [mg]: mean (*SD*) | 52.8 (99.4) |
| Eating before napping [Yes]: *n* (%) | 21 (20.0) |
| Listening to music during napping [Yes]: *n* (%) | 10 (9.5) |
| **Working environment** |  |
| Steps before napping per hour^a^ [steps/hour]: mean (*SD*) | 829.4 (219.4) |
| Number of hospitalized patients [person]: mean (*SD*) | 41.1 (5.6) |
| Number of patients each nurse responsible for [person]: mean (*SD*) | 10.3 (1.5) |
| Have a leadership role [Yes]: *n* (%) | 26 (24.8) |
| Event occurrence [Yes]: *n* (%) | 39 (37.1) |
| **Mood States (POMS2)** |  |
| AH (anger–hostility): mean (*SD*) | 2.6 (4.1) |
| CB (confusion-bewilderment): mean (*SD*) | 2.7 (3.0) |
| DD (depression–dejection): mean (*SD*) | 1.7 (2.8) |
| FI (fatigue-inertia): mean (*SD*) | 6.2 (4.4) |
| TA (tension–anxiety): mean (*SD*) | 4.3 (4.1) |
| VA (vigor-activity): mean (*SD*) | 2.2 (2.5) |
| F (friendliness): mean (*SD*) | 6.1 (3.1) |
| Total Mood Disturbance: mean (*SD*) | 15.3 (15.6) |
| **Arousal level (KSS)** |  |
| Start of the night shift: mean (*SD*) | 3.7 (1.5) |
| Before nap breaks: mean (*SD*) | 5.9 (1.6) |
| Change between start of the night shift to before nap breaks: mean (*SD*) | 2.2 (2.0) |
| **Intention to nap**: *n* (%) |  |
| Deep | 70 (66.7) |
| Light | 35 (33.3) |
| **Sleep-related status** |  |
| Start time of main sleep before the night shift [h:m]: mean (*SD*) | 0:26 (1:34) |
| End time of main sleep before the night shift [h:m]: mean (*SD*) | 9:16 (2:09) |
| Main sleep duration before the night shift [hour]: mean (*SD*) | 8.8 (1.8) |
| Prophylactic nap [Yes]: *n* (%) | 39 (37.1) |
| Total sleep duration before the night shift [hour]: mean (*SD*) | 9.4 (1.8) |
| Last awakening time before the night shift [h:m]: mean (*SD*) | 11:21 (2:32) |
| Awakening duration until nap breaks [hour]: mean (*SD*) | 13.8 (2.9) |

The sample units in this table are the number of night shifts. Night shifts in which nurses did not intend to nap were excluded.

Abbreviation: dB *L*_Aeq, napping_ decibel equivalent A-weighted sound pressure level during napping, KSS Karolinska Sleepiness Scale, POMS2 Profile of Mood States Second edition, *SD* standard deviation.
^a^ *n* = 104.
