# Supplementary material for: Quantity and quality of napping to mitigate fatigue and sleepiness among nurses working long night shifts: a prospective observational study
Source: J Physiol Anthropol. 2025 Jan 6;44:1. doi: 10.1186/s40101-024-00378-z (PMC11702087; doi:10.1186/s40101-024-00378-z)
Supplement: Supplementary file 4 — Additional file 4. Comparison of fatigue at the end of the night shift between combined TIB and SE groups. [file 40101_2024_378_MOESM4_ESM.docx]

**Additional file 4** Comparison of fatigue at the end of the night shift between combined TIB and SE groups

Fatigue at the end of the night shift (LS Mean [95%CI])

|  | | TIB (Time in Bed) | | |
| --- | --- | --- | --- | --- |
|  | | < 120 min | 120–180 min | > 180 min |
| SE (Sleep efficiency) | ≥ 70% | 52.4 [41.8, 63.0] | 45.7 [37.8, 53.5] | 45.2 [37.2, 53.3] |
|  | < 70% | 58.6 [50.0, 67.3] | 47.4 [39.4, 55.4] | 52.4 [43.8, 61.0] |

Multiple Comparisons

| **Group1** | **Group2** | **MD [95%CI]**  **(Group1 - Group2)** | **SE** | ***t*** | ***p*** |
| --- | --- | --- | --- | --- | --- |
| TIB > 180 min & SE ≥ 70% | TIB 120–180 min & SE ≥ 70% | -0.4 [-4.9, 4.1] | 2.3 | -0.19 | .854 |
|  | TIB < 120 min & SE ≥ 70% | -7.1 [-15.5, 1.2] | 4.2 | -1.71 | .093 |
|  | TIB > 180 min & SE < 70% | -7.2 [-12.5, -1.8] | 2.7 | -2.69 | .009 |
|  | TIB 120–180 min & SE < 70% | -2.2 [-6.5, 2.1] | 2.1 | -1.01 | .317 |
|  | TIB < 120 min & SE < 70% | -13.4 [-19.8, -7.0] | 3.2 | -4.18 | < .001 |
| TIB 120–180 min & SE ≥ 70% | TIB < 120 min & SE ≥ 70% | -6.7 [-14.5, 1.1] | 3.9 | -1.74 | .089 |
|  | TIB > 180 min & SE < 70% | -6.7 [-11.7, -1.7] | 2.5 | -2.71 | .009 |
|  | TIB 120–180 min & SE < 70% | -1.7 [-6.0, 2.5] | 2.1 | -0.82 | .414 |
|  | TIB < 120 min & SE < 70% | -13.0 [-18.8, -7.1] | 2.9 | -4.47 | < .001 |
| TIB < 120 min & SE ≥ 70% | TIB > 180 min & SE < 70% | 0.0 [-8.7, 8.7] | 4.4 | -0.01 | .996 |
|  | TIB 120–180 min & SE < 70% | 5.0 [-3.1, 13.0] | 4.0 | 1.24 | .222 |
|  | TIB < 120 min & SE < 70% | -6.2 [-15.4, 2.9] | 4.6 | -1.37 | .176 |
| TIB > 180 min & SE < 70% | TIB 120–180 min & SE < 70% | 5.0 [0.2, 9.8] | 2.4 | 2.10 | .040 |
|  | TIB < 120 min & SE < 70% | -6.2 [-13.0, 0.6] | 3.4 | -1.83 | .073 |
| TIB 120–180 min & SE < 70% | TIB < 120 min & SE < 70% | -11.2 [-17.2, -5.2] | 3.0 | -3.78 | < .001 |

Night shifts in which nurses did not intend to nap were excluded. The least squares means were estimated using the mixed-effects model for repeated measures, while post hoc *t*-tests were conducted using their estimates to calculate MDs between groups.

Abbreviation: CI confidence interval, LS least squares, MD mean difference, SE sleep efficiency, TIB time in bed.
