# Supplementary material for: Quantity and quality of napping to mitigate fatigue and sleepiness among nurses working long night shifts: a prospective observational study
Source: J Physiol Anthropol. 2025 Jan 6;44:1. doi: 10.1186/s40101-024-00378-z (PMC11702087; doi:10.1186/s40101-024-00378-z)
Supplement: Supplementary file 5 — Additional file 5. Comparison of sleepiness at the end of the night shift between combined TIB and SE groups. [file 40101_2024_378_MOESM5_ESM.docx]

**Additional file 5** Comparison of sleepiness at the end of the night shift between combined TIB and SE groups

Sleepiness at the end of the night shift (LS Mean [95%CI])

|  | | TIB (Time in Bed) | | |
| --- | --- | --- | --- | --- |
|  | | < 120 min | 120–180 min | > 180 min |
| SE (Sleep efficiency) | ≥ 70% | 3.3 [1.6, 4.9] | 4.2 [3.4, 5.1] | 4.7 [3.8, 5.6] |
|  | < 70% | 5.7 [4.4, 7.0] | 5.5 [4.7, 6.3] | 5.6 [4.6, 6.6] |

Multiple Comparisons

| **Group1** | **Group2** | **MD [95%CI]**  **(Group1 - Group2)** | **SE** | ***t*** | ***p*** |
| --- | --- | --- | --- | --- | --- |
| TIB > 180 min & SE ≥ 70% | TIB 120–180 min & SE ≥ 70% | 0.5 [-0.6, 1.5] | 0.5 | 0.91 | .367 |
|  | TIB < 120 min & SE ≥ 70% | 1.5 [-0.3, 3.2] | 0.9 | 1.69 | .101 |
|  | TIB > 180 min & SE < 70% | -0.9 [-2.1, 0.4] | 0.6 | -1.37 | .177 |
|  | TIB 120–180 min & SE < 70% | -0.8 [-1.8, 0.2] | 0.5 | -1.52 | .134 |
|  | TIB < 120 min & SE < 70% | -1.0 [-2.5, 0.6] | 0.8 | -1.25 | .217 |
| TIB 120–180 min & SE ≥ 70% | TIB < 120 min & SE ≥ 70% | 1.0 [-0.7, 2.6] | 0.8 | 1.21 | .235 |
|  | TIB > 180 min & SE < 70% | -1.3 [-2.5, -0.2] | 0.5 | -2.46 | .019 |
|  | TIB 120–180 min & SE < 70% | -1.3 [-2.2, -0.3] | 0.5 | -2.67 | .010 |
|  | TIB < 120 min & SE < 70% | -1.4 [-2.8, -0.1] | 0.7 | -2.15 | .038 |
| TIB < 120 min & SE ≥ 70% | TIB > 180 min & SE < 70% | -2.3 [-4.2, -0.4] | 0.9 | -2.47 | .018 |
|  | TIB 120–180 min & SE < 70% | -2.2 [-3.9, -0.5] | 0.8 | -2.64 | .012 |
|  | TIB < 120 min & SE < 70% | -2.4 [-4.4, -0.4] | 1.0 | -2.42 | .019 |
| TIB > 180 min & SE < 70% | TIB 120–180 min & SE < 70% | 0.1 [-1.0, 1.1] | 0.5 | 0.17 | .864 |
|  | TIB < 120 min & SE < 70% | -0.1 [-1.7, 1.5] | 0.8 | -0.11 | .911 |
| TIB 120–180 min & SE < 70% | TIB < 120 min & SE < 70% | -0.2 [-1.6, 1.2] | 0.7 | -0.25 | .804 |

Night shifts in which nurses did not intend to nap were excluded. The least squares means were estimated using the mixed-effects model for repeated measures, while post hoc *t*-tests were conducted using their estimates to calculate MDs between groups.

Abbreviation: CI confidence interval, LS least squares, MD mean difference, SE sleep efficiency, TIB time in bed.
