# Supplementary material for: Quantity and quality of napping to mitigate fatigue and sleepiness among nurses working long night shifts: a prospective observational study
Source: J Physiol Anthropol. 2025 Jan 6;44:1. doi: 10.1186/s40101-024-00378-z (PMC11702087; doi:10.1186/s40101-024-00378-z)
Supplement: Supplementary file 6 — Additional file 6. Comparison of Δfatigue from start to end of the night shift between combined TIB and SE groups. [file 40101_2024_378_MOESM6_ESM.docx]

**Additional file 6** Comparison of Δfatigue from start to end of the night shift between combined TIB and SE groups

Δfatigue from start to end of the night shift (LS Mean [95%CI])

|  | | TIB (Time in Bed) | | |
| --- | --- | --- | --- | --- |
|  | | < 120 min | 120–180 min | > 180 min |
| SE (Sleep efficiency) | ≥ 70% | 18.4 [7.3, 29.6] | 2.9 [-1.5, 7.4] | 6.0 [1.2, 10.8] |
|  | < 70% | 12.3 [6.5, 18.0] | 7.2 [3.3, 11.2] | 9.8 [3.9, 15.6] |

Multiple Comparisons

| **Group1** | **Group2** | **MD [95%CI]**  **(Group1 - Group2)** | **SE** | ***t*** | ***p*** |
| --- | --- | --- | --- | --- | --- |
| TIB > 180 min & SE ≥ 70% | TIB 120–180 min & SE ≥ 70% | 3.1 [-3.0, 9.2] | 3.1 | 1.00 | .321 |
|  | TIB < 120 min & SE ≥ 70% | -12.4 [-24.7, -0.1] | 6.1 | -2.03 | .048 |
|  | TIB > 180 min & SE < 70% | -3.8 [-10.8, 3.3] | 3.5 | -1.08 | .285 |
|  | TIB 120–180 min & SE < 70% | -1.2 [-6.9, 4.4] | 2.8 | -0.43 | .667 |
|  | TIB < 120 min & SE < 70% | -6.3 [-13.7, 1.2] | 3.7 | -1.69 | .097 |
| TIB 120–180 min & SE ≥ 70% | TIB < 120 min & SE ≥ 70% | -15.5 [-27.1, -3.9] | 5.8 | -2.69 | .010 |
|  | TIB > 180 min & SE < 70% | -6.8 [-13.6, 0.0] | 3.4 | -2.03 | .049 |
|  | TIB 120–180 min & SE < 70% | -4.3 [-9.9, 1.3] | 2.8 | -1.53 | .130 |
|  | TIB < 120 min & SE < 70% | -9.3 [-16.2, -2.4] | 3.4 | -2.71 | .009 |
| TIB < 120 min & SE ≥ 70% | TIB > 180 min & SE < 70% | 8.6 [-3.6, 20.9] | 6.1 | 1.42 | .162 |
|  | TIB 120–180 min & SE < 70% | 11.2 [-0.3, 22.7] | 5.7 | 1.97 | .056 |
|  | TIB < 120 min & SE < 70% | 6.1 [-6.0, 18.3] | 6.0 | 1.02 | .311 |
| TIB > 180 min & SE < 70% | TIB 120–180 min & SE < 70% | 2.6 [-4.1, 9.2] | 3.3 | 0.77 | .443 |
|  | TIB < 120 min & SE < 70% | -2.5 [-10.6, 5.6] | 4.0 | -0.62 | .540 |
| TIB 120–180 min & SE < 70% | TIB < 120 min & SE < 70% | -5.0 [-11.4, 1.4] | 3.2 | -1.59 | .120 |

Night shifts in which nurses did not intend to nap were excluded. The least squares means were estimated using the mixed-effects model for repeated measures, while post hoc *t*-tests were conducted using their estimates to calculate MDs between groups.

Abbreviation: CI confidence interval, LS least squares, MD mean difference, SE sleep efficiency, TIB time in bed.
